# Reproductive Biology of Three Important Threatened/Near-Threatened Groupers (*Plectropomus leopardus*, *Epinephelus polyphekadion* and *Plectropomus areolatus*) in Eastern Indonesia and Implications for Management

**DOI:** 10.3390/ani9090643

**Published:** 2019-09-02

**Authors:** Miftakhul Khasanah, Nadiarti Nurdin Kadir, Jamaluddin Jompa

**Affiliations:** Faculty of Marine Science and Fisheries, Hasanuddin University, Jl. Perintis Kemerdekaan km. 10, Makassar 90245, Indonesia

**Keywords:** sustainable fisheries management, threatened groupers, spawning aggregations, size at first maturity, reproductive patterns

## Abstract

**Simple Summary:**

Indonesia needs basic data on the reproductive dynamics of economically important fishery species, including groupers, in order to support sustainable fisheries management. Data from the histological examination of grouper gonad samples were combined with data from: participatory mapping and interviews with fishermen, monitoring data from known spawning aggregation sites, and analyzed descriptively to provide basic data on the size at first maturity and seasonal reproductive patterns of three economically important groupers. We suggest management measures including size limits, temporal spatial closures, and a trading ban during the peak spawning season (November–December).

**Abstract:**

The three grouper species most heavily fished for the live reef fish trade (LRFT) in Indonesia are *Plectopomus leopardus* (greatest catch volume), and two species classified as Vulnerable on the International Union for Conservation of Nature (IUCN) Red List: *Plectropomus areolatus* and *Epinephelus polyphekadion*. Understanding the reproductive biology of these fishes is essential for sustainable management, but relevant data are limited. This study aimed to determine reproductive dynamics, so as to inform management measures to maintain the reproductive capacity of these groupers. Grouper gonad samples collected from fish caught for the LRFT were analyzed histologically. Data were also collected from participatory mapping and interviews with fishermen, and underwater monitoring of three known spawning aggregation sites in the Wakatobi National Park, Eastern Indonesia. Based on observed gonad development, the respective lengths and weights at first maturity were: 37.7 cm and 759 g (*P. leopardus*); 36.65 cm and 771.2 g (*P. areolatus*); 36.95 cm and 889.9 g (*E. polyphekadion*). The mean weight of the groupers market-based sampled was higher than the size at first sexual maturity. Sex transition was observed in *P. leopardus*; sex reversal was not observed in *E.* *polyphekadion*, and the sex pattern of *P. areolatus* was unresolved. Based on the fisher surveys and spawning aggregation monitoring, spawning occurs around the new moon from September to April, with reproductive peaks in November and December. Fisheries management measures that are suggested to sustain grouper stocks include enforcing appropriate size limits, temporal spatial closures (spawning aggregation sites), and a trading ban during the peak spawning season (November–December).

## 1. Introduction

Indonesia is a major exporter of live reef fishes, especially groupers [1]. Various rules and regulations at national and provincial levels have been put in place; however, these regulations are poorly enforced and inadequate to support the sustainable management of these species [2]. High profits in the live reef food fish trade and weak surveillance by law enforcement officers motivate Indonesian fishers and traders to avoid compliance, encouraging the continued use of destructive fishing practices [3]. Consequently, excessive extraction levels are prevalent and widely reported [4]. Negative impacts from overfishing and destructive practices affect grouper stocks directly and indirectly through the degradation of coral reefs and their capacity to provide grouper habitat [5].

The leopard coral trout (*Plectropomus leopardus*), squaretail coral grouper (*Plectropomus areolatus*) and camouflage grouper (*Epinephelus polyphekadion*) are three of the 10 fishes most commonly sought by the live reef fish trade (LRFT) in Southeast Asia [6,7]. These fishes have been intensively exploited throughout Indonesia over the past four decades [8]. The leopard coral trout contributes the highest volume to LRFT exports, followed by the squaretail coral grouper and camouflage grouper [7]. The squaretail coral grouper and camouflage grouper are relatively fast-growing species [9,10], maturing early; however, the spawning aggregations of these species are easy to find and usually well known to fishers, making these groupers especially vulnerable to overfishing [10,11,12,13,14]. The high demand for leopard coral trout was initially prompted by its attractive body coloration (red skin and white meat), making this grouper a menu of choice for Chinese banquets, especially on the eve of the Chinese New Year, as the color red is a symbol of good fortune in Chinese culture [15,16,17]. Heavy exploitation of spawning aggregations can pose a severe threat to marine fishes, affecting reproductive success and the long-term sustainability of capture fisheries, and can eventually lead to the extirpation of fish stocks and cessation of aggregation at overfished sites [18,19]. Based on the dramatic reduction in fish populations due to excessive extraction, the IUCN Red List assessments designated *P*. *areolatus* and *E. polyphekadion* as Vulnerable (VU A2bd) in 2018 [20,21]. These assessments state that steps to improve the management of these species are urgently needed. Although the IUCN Red List assessment placed *P*. *leopardus* in the Least Concern (LS) category [22], the management of this species needs to be improved in order to prevent it qualifying for Vulnerable (or even Endangered) status. Although a number of studies from several countries have been conducted on the reproductive dynamics of these species [5,9,10,23,24], data on sexual patterns and spawning seasons in Indonesia are still lacking [9].

Size limitation is one of the least complicated fisheries management approaches to dealing with overfishing and is relatively straightforward to implement [25]. Most countries with a LRFT fishery (with the exception of Australia) lack regulations for the target species [9]. Australia has enacted regulations on allowable catch size, restrictions on fishing gear, recreational bag limits, and spatial–temporal closures for several groupers, including *P. leopardus* [15]. In practice, some live grouper traders in Indonesia have set a 500 g minimum weight limit for the live groupers which they purchase for the LRFT (Hj Said, *personal communication*, 18 April 2017). However, this weight limit does not as yet have any legal basis; furthermore, there is some uncertainty as to the likely effectiveness of this size limit (500 g), due to insufficient data on the average size of the fish caught and reproductive dynamics, including size at first maturity and spatial and temporal data on spawning aggregations [9,26]. Therefore, it is important to understand the reproductive dynamics of the three most commonly traded groupers in the Indonesian LRFT (*P. leopardus*, *E. polyphekadion*, and *P. areolatus*) as a basis for sustainable fisheries management approaches.

This study aimed to determine important reproductive parameters (minimum size at first maturity, evidence of sex change and spawning seasons) to support sustainable fisheries management of three important highly exploited (including two near-threatened) groupers (*P. leopardus*, *E. polyphekadion*, and *P. areolatus*) in Eastern Indonesia. The data on reproductive biology were used to formulate recommendations regarding potential fisheries management approaches for the studied grouper species (leopard coral trout, squaretail coral grouper, and camouflage grouper) in Eastern Indonesia.

## 2. Materials and Methods

**Figure 1:**
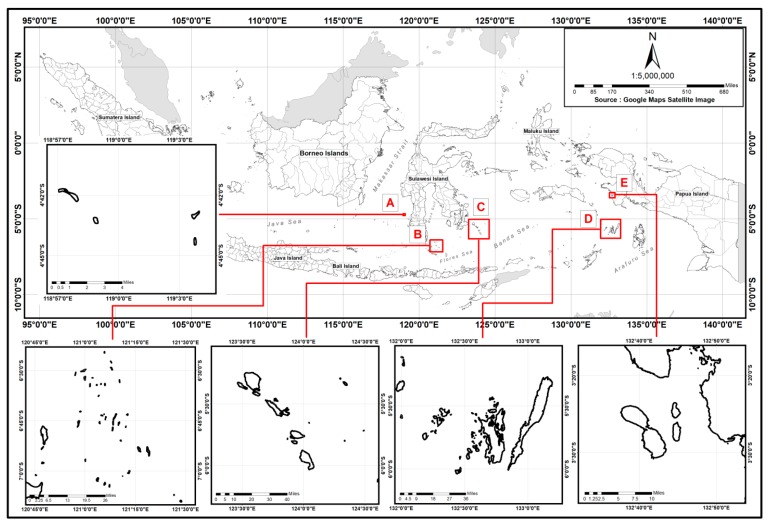
Site maps of the study areas for gonad sampling and spawning aggregation site mapping; (**A**) Kapoposang Marine Tourism Park, (**B**) Takabonerate National Park, (**C**) Wakatobi National Park, (**D**) Kei Islands, and (**E**) Karas Islands, Fak-Fak Regency.

Data on the weight (g) of traded groupers were collected from one private company (2015–2016) for leopard coral trout and squaretail coral grouper. Data on the weight of camouflage grouper were collected during 2016 from 5000 fishers in 61 locations within Eastern Indonesia (from East Kalimantan to Papua). Gonads of the three species studied (leopard coral trout, squaretail coral grouper, and camouflage grouper) were collected from groupers caught by hook and line during the reproductive season (between 24 October 2017, and 4 April 2018) in five areas within Eastern Indonesia. These areas were Kapoposang Marine Tourism Park, Takabonerate National Park, Wakatobi National Park, Kei Islands and Karas Islands (Figure 1). The reproductive season for the three groupers was estimated based on previous studies. The leopard coral trout is reported to spawn around the new moon during September–November across much of its wide Indo-Pacific distribution, including the Takabonerate Archipelago [9,26]. Squaretail coral grouper are reported as forming large spawning aggregations during September–February in Selayar [9,26], as well as February–May [10] around the third quarter or new moon phase in Pohnpei [20]. Camouflage grouper spawning has been reported during October–November and January–March in Sinjai [26].

The gonads of the sampled groupers were extracted, weighed (±0.1 g) and preserved in 10% formalin. Gonad samples were transported by sea and airfreight from the sampling locations to the laboratory of the Balai Besar Veteriner, Maros, in South Sulawesi. Histological slides were prepared as follows. Tissue was excised from a central portion of one gonad lobe of each gonad. The samples were subsequently dehydrated through a series of increasing concentrations of ethanol, cleared in xylene, infiltrated, and embedded in paraffin wax. Transverse sections of 7 µm, made using a hand rotary microtome, were mounted on glass slides using Mayer’s egg albumin. The samples were then rehydrated and stained with Harris hematoxylin, and counterstained with eosin. Both ovaries and testes were examined under a light microscope in the School of Biological Science, at The University of Hong Kong.

The histological structure of gonads extracted from the three studied groupers species was similar to that described for the Nassau grouper (*Epinephelus striatus*) (Table 1) [27]. Therefore, assignment of sexual categories and maturity development was based on a modification of the criteria developed for the Nassau grouper (*Epinephelus striatus*) [27]. The gonads were then classified as immature, or as one of four stages of sexual maturation, from ‘resting’ to ‘post-spawning’ (Table 1). When gonad tissue contained male (testes or seminiferous tubules) and female (ovarian gonadal structures include lamellae, lumen and numerous stages of oocytes of gonad maturation) characteristics, the gonad was classified as ‘bisexual’; however, such characteristics could be unrelated to hermaphroditism [27,28,29]. Data on gonad development was limited to gonad morphology and features providing evidence of ‘sexual change’ in the samples, and were not related to sexual function [27,29].

Whenever possible, the total length (TL in cm) and weight (whole body mass, W in g) of the groupers were measured. These data were used to calculate the length–weight relationship (W = *a*L*^b^*) parameters *a* and *b*. In the live reef food fish trade (LRFFT) in Indonesia, fish size (for buying and selling and for reporting) is commonly based on weight (whole body size), not length. The mean size at first maturity (Lm_50_) for female groupers of each species was estimated by fitting a log transformed regression function to the proportion of mature fish in each size cohort [30]. Because live groupers are typically measured by weight, while the regression used requires length, the weight data were converted to length using the length-weight relationship equation W = *a*L*^b^*, where W is fish bodyweight (g), L is total length (cm), *a* means a constant obtained from the intercept of the regression model, and *b* is the regression coefficient.

Participatory mapping interviews with the target of 50 fishers per site, for 5 sites, were used to obtain an initial indication of grouper spawning seasons at each site, as suggested by the spawning aggregation sites (SPAGs) monitoring manual from the Society for the Conservation of Reef Fish Aggregation (SCRFA) guidebook [31]. The respondents were asked questions regarding any evidence of spawning aggregation sites, types of grouper, spawning periods, water depth at spawning sites, and any changes in spawning aggregations over time. Interviews were conducted with fishers from different age groups and generations (Appendix A).

Additional data on spawning seasons were obtained from the Wakatobi National Park Authority surveys conducted during spawning seasons from 2005–2011 under a joint monitoring program (2005–2011) by The Nature Conservancy (TNC), the World Wild Fund (WWF) and the Wakatobi National Park Authority. Three spawning aggregation sites of the squaretail coral grouper were monitored using the methods in the SCRFA guidebook [31]. Underwater visual census surveys were conducted at least once every two months with each survey lasting one week starting from the waning gibbous moon phase. The surveyors collected data on species identity and fish behavior over the spawning period, such as aggression, courtship, visibly gravid fish, and spawning activity. They also estimated the sizes (total length) of individual fish and number of fish per species in each aggregation.

## 3. Results

### 3.1. Histological Characteristics of Gonad Developmental Stages

The sampled leopard coral trout gonads examined microscopically came from wild grouper caught in the study areas by fishermen, as follows: Kapoposang Marine Tourism Park (MTP) (*n* = 34), Takabonerate National Park (NP) (*n* = 18), Karas Islands (*n* = 31), and Kei Islands (*n* = 5). Squaretail coral trout gonad samples were collected from the Takabonerate National Park (*n* = 9), Wakatobi National Park (*n* = 15), and Karas Islands (*n* = 1). Camouflage grouper samples were also collected from the Takabonerate National Park (*n* = 15) and Wakatobi National Park (*n* = 5) (Table 2).

#### 3.1.1. Leopard Coral Trout (*Plectropomus leopardus*)

**Figure 2:**
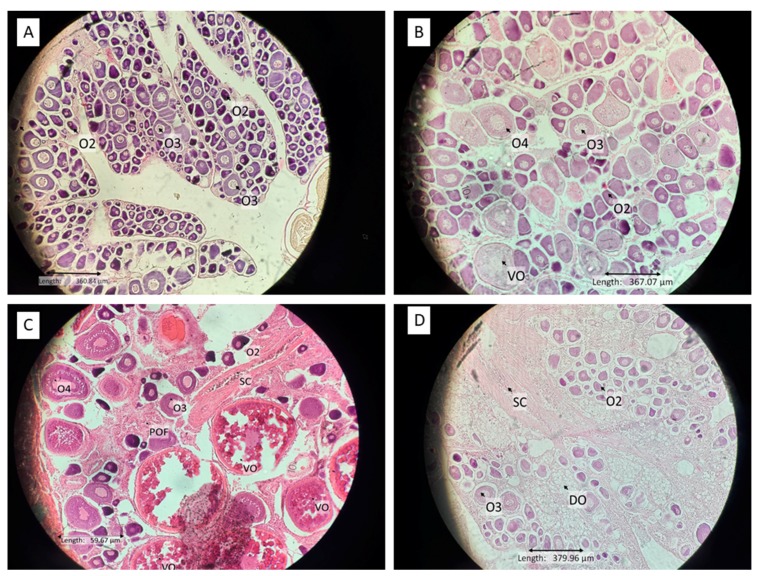
Histological slides of grouper ovaries. (**A**) Immature female leopard coral trout, collected 12 December 2017, total length (TL) = 35 cm; (**B**) Mature resting female leopard coral trout collected 12 December 2017, TL = 45 cm; (**C**) Mature ripe female squaretail coral trout collected 21 December 2017, TL = 43 cm; (**D**) Post spawning female camouflage grouper collected 11 November 2017, TL = 47 cm; O2, stage1/2 oocytes; O3, stage 3 oocytes; O4, stage 4 oocytes; VO, vitellogenic oocyte; DO, degenerated oocyte; POF, postovulatory follicles; SC, spermatocytes.

**Figure 3:**
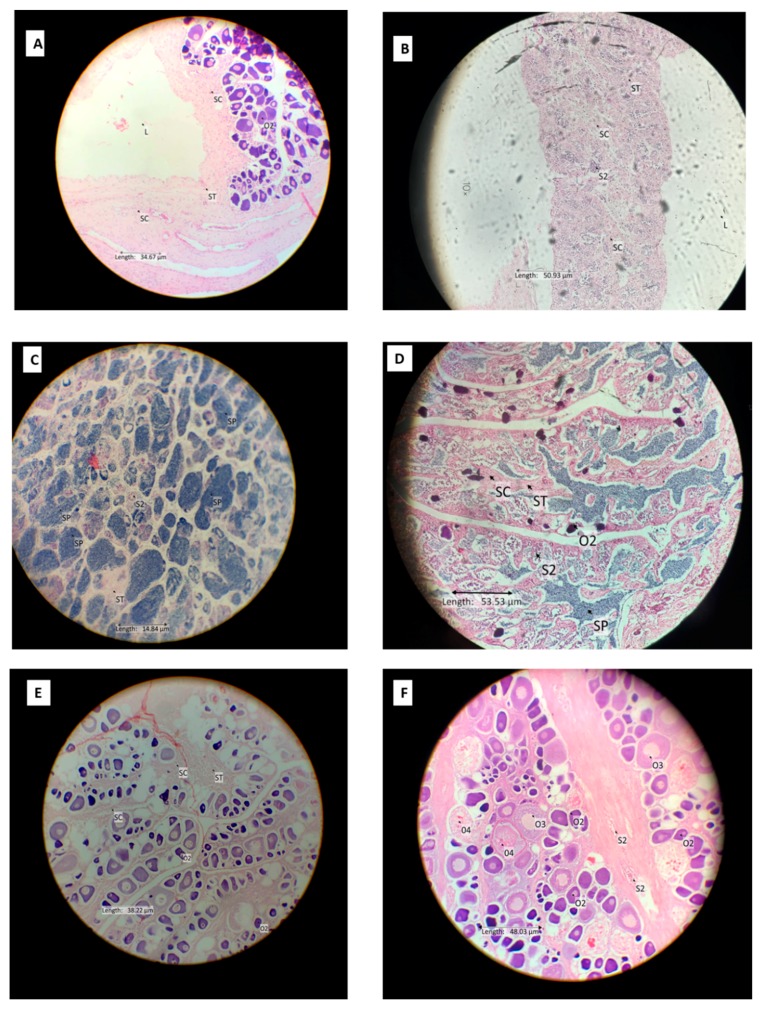
Histological slides of grouper testes and intersex gonads. (**A**) Immature male camouflage grouper collected 26 November 2017, TL = 39 cm; (**B**) Mature resting male leopard coral trout collected 7 January 2018, TL = 45 cm; (**C**) Mature ripe male leopard coral trout collected 22 February 2018, TL = 60 cm; (**D**) Post spawning male leopard coral trout collected 20 February 2018, TL = 56 cm; (**E**) Immature female dominant bisexual leopard coral trout collected 10 November 2017, TL = 27 cm; (**F**) Mature bisexual leopard coral trout collected 27 December 2017, TL = 42 cm; S2, stage 2 spermatocytes; SC, stage 1 oocytes; SP, spermatid/spermatozoa; ST, seminiferous tubules.

All stages of leopard coral trout gonad maturation development were observed during this research with the exception of immature males. Reproductively inactive females had previtellogenic oocytes and included immature and resting mature females (Figure 2A,B). The cell wall was still somewhat irregular in shape (but less so than oocyte stage I), and the nucleus began to be apparent with few or many pale spots in the lipid vesicles within. In mature active females, the oocyte cells contained vitellin globules, which stained moderately with eosin. The oocytes were double in size in the late mature stage, and the cell walls thickened. The vitellogenic oocytes become hydrated in the post-spawning stage. Male stage features were found in the gonads of larger coral trout specimens, some inactive primary stage oocytes 1/2 were recovered in this stage. Spermatocytes began to fill the lumen in the mature stage. After spawning, there were empty spaces in the lumen of male coral trout gonads (Figure 3C,D). The bisexual phase was recorded in both small and large leopard coral trout size classes. Mature bisexual gonads contained stage 2 spermatocytes combined with a high abundance of stage 1/2, 3 oocytes (Figure 3F). This condition is likely to be related to the transition stage from female to male, indicating that this species may be protogynous.

**Figure 4:**
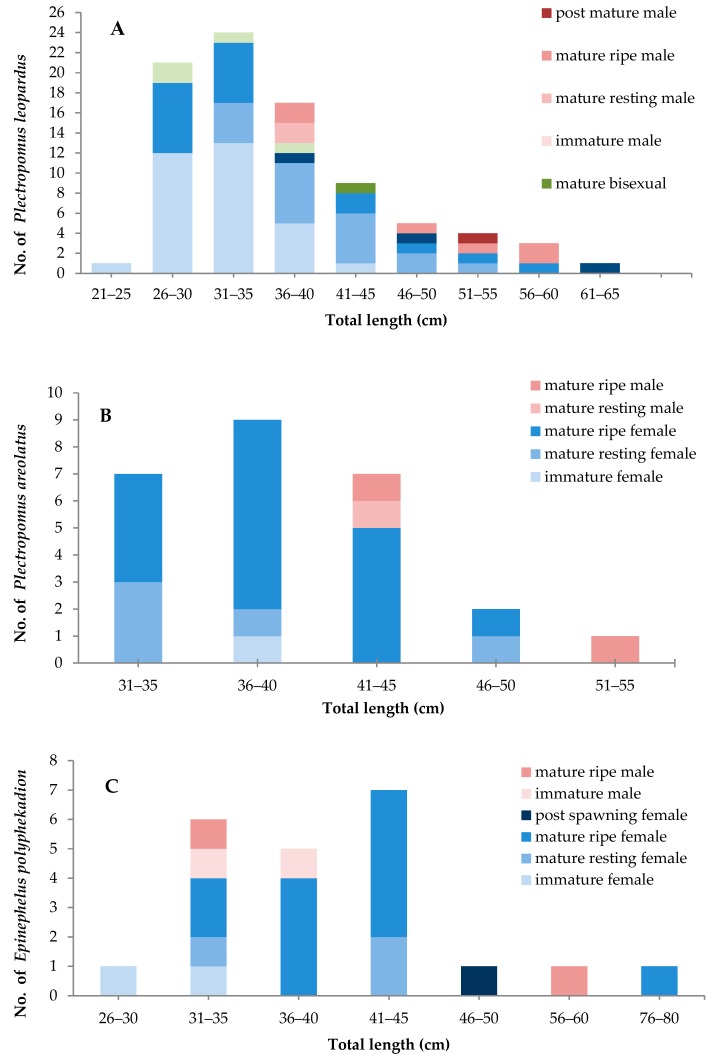
Stages of gonad maturity of leopard coral trout by size class (TL) (**A**), squaretail coral trout (**B**), and camouflage grouper (**C**).

Sexual patterns, sex ratio, and approximate mean size of sexual maturation in the leopard coral trout were determined from the limited samples (*n* = *85*) observed in this study (Figure 4A). The smallest sampled female with inactive phase gonads measured 24 cm TL and the smallest mature, ripe female measured 27 cm TL. The smallest mature, ripe male measured 36 cm TL. Coral trout in the smaller size classes, but with mature gonad development stages, were mostly from the Kapoposang MTP. For both male and female leopard coral trout, mature active (ripe) gonads were first observed in fish caught during October and continued until December, with the largest proportion of mature active fish observed in November (around 40% of all samples with mature gonads). Inactive females and males (mature, resting) were most common in December (50% of all mature, resting samples). In the leopard coral trout sampled, most inactive female gonads (immature and resting) were found in large individuals (42–51 cm TL). The histological data indicate that males have two pathway developments: direct from juvenile phase to male, or via sex change from adult female. These observations indicate diandric protogyny; however, due to the relatively small sample size, further sampling is needed to confirm whether this is indeed the case.

#### 3.1.2. Squaretail Coraltrout (*Plectropomus areolatus*)

The number of squaretail coral trout gonad samples (*N* = 24) was low, but covered a wide range of size classes, enabling many stages of gonad development to be observed (Figure 4B). Inactive female gonads contained the primary 1/2 and 3 stage oocytes. Mature active ripe female gonads contained a high abundance of vitellogenic oocytes; post-ovulatory follicles were observed at this stage, indicating spawning within the previous 24 h (Figure 2C). Based on the interviews with fishermen, this species aggregates to spawn during daylight hours, beginning around noon and peaking in the late afternoon, from around 4 p.m. until dusk. The length of the smallest reproductively inactive female was 21 cm TL, and that of the smallest mature, ripe female was 33 cm TL. The smallest mature, ripe male measured 42 cm TL. There was no evidence of bisexuality at any gonad development stage, even in the larger-sized fish. The peak season based on gonad maturity was in December (50% of all mature gonad samples) for both males and females. The sexual pattern for this species is unresolved. Therefore, further research is urgently needed.

#### 3.1.3. Camouflage grouper (*Epinephelus polyphekadion*)

Reproductively inactive, but clearly female and male, camouflage grouper were observed (*n* = 22) in the small size class (27–31 cm) TL (Figure 4C). The smallest reproductively immature female was 21 cm TL in length, while the smallest mature active female measured 34 cm TL. The smallest immature male was 32 cm TL, and the smallest mature active male was 34 cm TL. This indicates that sexual differentiation tends to occur before the onset of gonad maturation, and sex reversal is unlikely. Although the number of fish sampled in this study was relatively low, the presence of immature males (and females) in the smaller size range (32–39 cm TL) and mature, resting female and males in substantially the same (larger) size range (34–80 cm TL for females and 34–55 cm TL for males) provides supporting evidence for an absence of sex reversal in this species. The highest percentage of mature fish (male and female, 50%) was observed in January.

### 3.2. Mean Size at Sexual Maturity

Length–frequency histograms of various maturity stages by sex (Figure 4.) showed that the mature gonad development phases dominated in the grouper size-classes represented in the study sample. In leopard coral trout and squaretail coral trout, male gonads (testes) were more common in the larger size classes. In contrast, camouflage grouper males were also common in the smaller size classes, with a similar size distribution to females. Based on the fitted regression curve for each of these three species, the total length at 50% sexual maturation (Lm_50_) varied between species (Table 3).

Based on the fishers’ interviews, the history of grouper fishing for the LRFT differs between the sampling sites, with some beginning to be exploited earlier than others. Hong Kong traders have been buying live reef fish caught in the Makassar Strait since the 1980s, including the Kapoposang Marine Tourism Park (MTP) site, followed by expansion to the Takabonerate National Park (NP) area and the Wakatobi (not yet a national park) around 1984. The trade continued to expand eastwards in Indonesia, reaching West Papua (Karas Islands) and the Southeast Moluccas (Kei Islands) in the 1990s. Each area has specific dominant grouper species; for example, the LRFT in Kapoposang and Kei is dominated by leopard coral trout, while in the Wakatobi and Takabonerate areas, the main species fished and traded are the squaretail coral trout and camouflage grouper. Table 3 shows that fish at the most extended places have been extracted, related to a smaller size at maturity.

**Figure 5:**
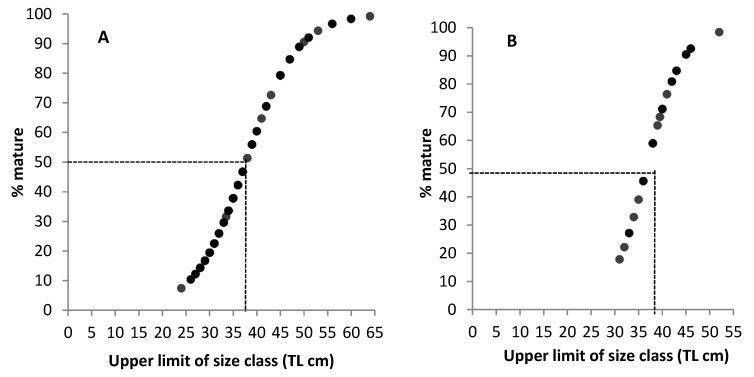

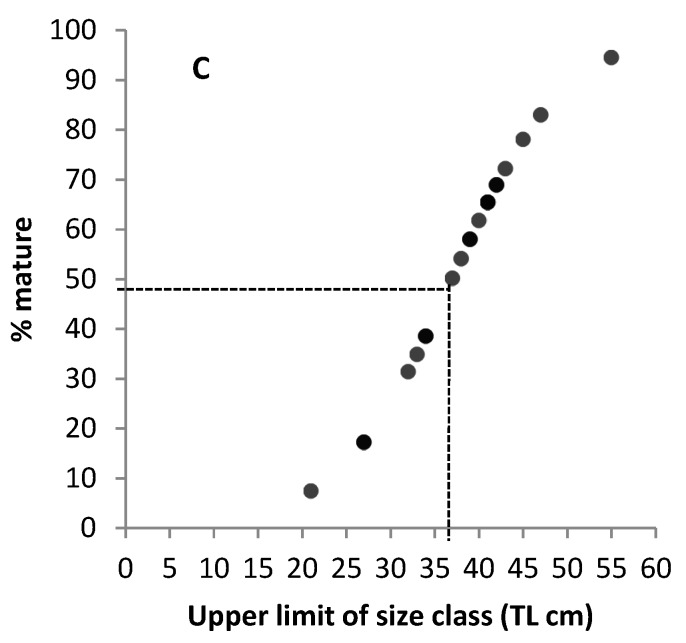
Length at 50% maturity (Lm_50_) estimated for leopard coral trout (**A**), squaretail coral trout (**B**), and camouflage grouper (**C**) from Eastern Indonesia during the spawning season (*n* = 76). Percentages reflect the proportion of mature females (mature resting, ripe, and post-spawning) per size class. The function is: y = 100/1 + e^−1(Xmid−X0.5)/d)^.

Based on the fitted regression curve applied to all specimens of each species (all sites pooled), the mean total lengths at 50% sexual maturation were as follows: for leopard coral trout Lm_50_ = 37.7 cm TL; for squaretail coral trout Lm_50_ = 36.6 cm TL; and for camouflage grouper Lm_50_ = 37.0 cm TL (Figure 5).

**Figure 6:**
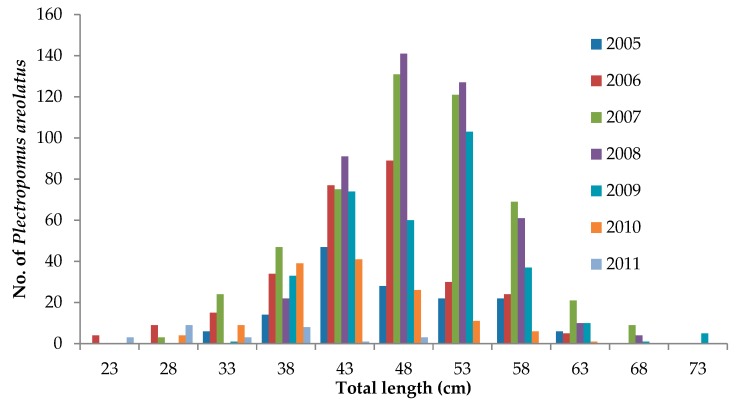
Estimated total length of *Plectropomus areolatus* observed at a known spawning aggregation site in Wakatobi National Park (*n* = 1876). Source: Data from the Wakatobi National Park Authority, 2017.

**Figure 7:**
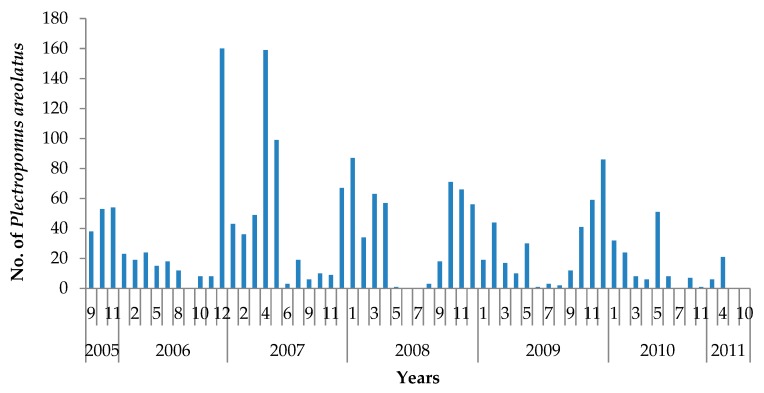
Count of individual *Plectropomus areolatus* in a known spawning aggregation site in Wakatobi National Park (*n* = 1876). Source: Data from the Wakatobi National Park Authority, 2017.

For squaretail coral trout, the length of fish aggregating to spawn during spawning seasons over the period 2005–2011 was confirmed by the underwater census data from known aggregating sites in the Wakatobi NP. In fact, several species of grouper were seen to aggregate at these sites, including *Epinephelus fuscoguttatus*, *E. polyphekadion*, *P. areolatus*, and *Lutjanus bohar*. The only species observed which did not show any evidence of spawning behavior (such as aggression, courtship, and visibly gravid fish) was *Epinephelus polyphekadion*. The mean size of mature squaretail coral trout estimated visually at the spawning sites ranged from 23 TL cm to 73 TL cm (Figure 6); the number of individual fish counted in one survey ranged from 2–92 individuals (Figure 7) (Data from the Wakatobi National Park Authority, 2017).

### 3.3. Spawning Seasons

**Figure 8:**
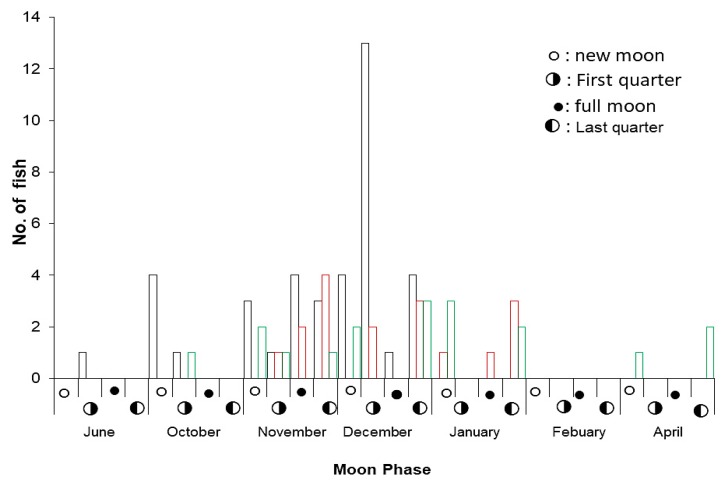
Mature fish (mature resting, ripe, and post-spawning) sampled June 2017–April 2018, by collection month and moon phase: leopard coral trout (black); squaretail coral trout (red); and camouflage grouper (green).

Based on the gonad development of the fish sampled, spawning appears to begin in late September and continues until early April, for all three grouper species in this study (Figure 8). Ripe male and female leopard coral trout were found in the last quarter moon phase in both November and December. Mature ripe female and male squaretail coral trout were found in the last quarter moon phase in January. Mature male and female camouflage grouper were found over several months, and this was the only species studied with indications of reproductive activity in April, around the new moon.

These data were supported by interviews with fishermen (*n* = 228). According to the respondents, leopard coral trout spawn during October to January; squaretail coral trout from September to May, and camouflage grouper from October to April (Table 4). Data from the Wakatobi National Park Authority monitoring show squaretail coral trout spawning around the new moon, from November–December and March–May (Figure 8).

### 3.4. Live Grouper Market Size

**Figure 9:**
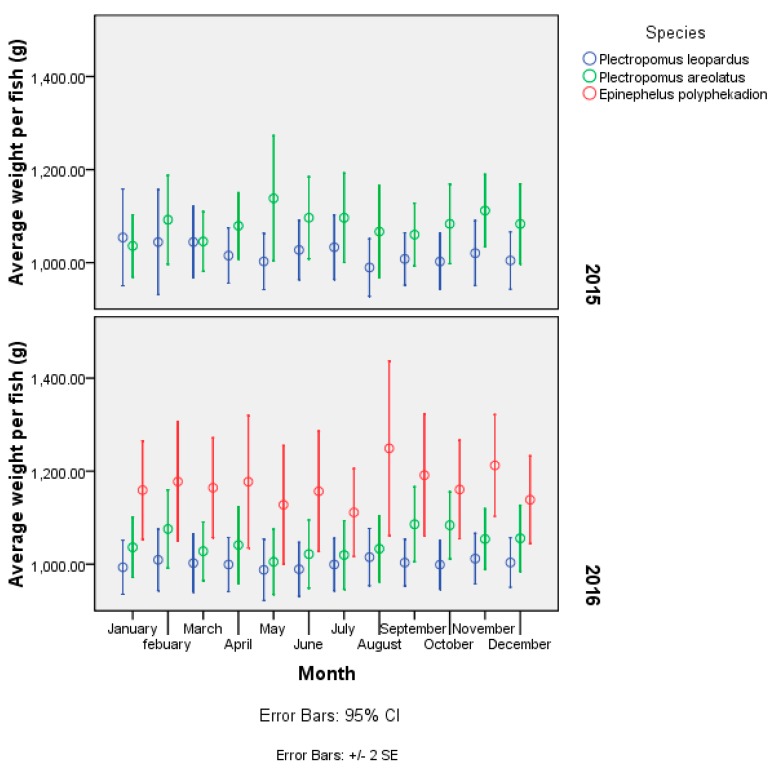
Data on mean weight (g) of *P. leopardus* (*n* = 2475), *P. areolatus* (*n* = 2182) and *E. polyphekadion* (*n* = 779) caught and traded in the live reef fish trade (LRFT) in Eastern Indonesia from (2015–2016).

The size of live groupers in the LRFT is commonly recorded as weight rather than length. Data on the weight of commercial catch from 2015–2016 show that weight per fish for *P. leopardus* ranged from 213–6000 g (*n* = 1249) in 2015 and 500–2900 g (*n* = 1226) in 2016. The bodyweight of *P. areolatus* ranged from 500–7086 g per fish in 2015, and 500–3300 g per fish in 2016, with a statistically significant decrease in mean weight from 1083 g (*n* = 1140) in 2015 to 1046 g (*n* = 1042) in 2016 (*T*-test, d.f = 2136, *p* < 0.025). *Epinephelus polyphekadion* is a relatively large species [24], and was on average significantly larger than leopard coral trout and *P. areolatus* (Kruskal–Wallis, d.f. = 2, *p* < 0.000), with a mean weight of 1170 g per fish (*n* = 799) and a range from 500 g up to 5350 g per fish in 2016 (Figure 9).

Based on the length–weight relationship conversion of the first length maturity of groupers, the mean weights at which these three species reach the first maturity are 759 g for *P. leopardus*, 771 g for *P. areolatus*, and 890 g for *E. polyphekadion*. Meanwhile the trade data show that the average sizes of traded fish are 892.3 g for *P. leopardus*; 975.3 g for *P. areolatus*; and 1051.3 g for *E. polyphekadion*. Thus, the mean size at first maturity is lower than the average size of fish in the commercial catch.

## 4. Discussion

### 4.1. Sexual Patterns

Evidence of sex transition (presence of male gonad tissue in the late female stage of gonad development) [29] was observed in a leopard coral trout, TL = 42 cm (Figure 5), but was not found in squaretail coral trout or camouflage grouper. Although the squaretail coral grouper males were in the large size classes, the sample size was not sufficient to draw any strong conclusion; the sexual pattern remains unresolved, but with strong suggestions of *hermaphrodite protogyny* [10]. The presences of small immature males in camouflage grouper indicate potential sexual differentiation before gonad maturation; in other words, it seems likely that the camouflage grouper does not undergo sex reversal. The sex pattern of the leopard coral trout is strongly suggested as *diandric protogyny* [33], meaning that male leopard coral trout can derive from immature or mature females. Most grouper species are known to change sex; the lower number of males compared to females in this research is consistent with the sex reversal processes. Considering that this is the first such research on these species in Eastern Indonesia, further research appears to be both warranted and urgent.

### 4.2. Spawning Period

Most groupers traded in the LRFT are intrinsically vulnerable to over-fishing, due to the large number of fish aggregating at predictable places and times [18]. This is known to be a particularly high risk factor for three grouper species (*P. leopardus*, *E. polyphekadion*, and *E. fuscoguttatus*) [12]. In this study, the spawning period of leopard coral trout lasted from October to January with a peak season in November and December around the last quarter moon phased, similar to the spawning period in Great Barrier Reef, Australia in September to December [33]. The spawning period of squaretail coral grouper lasts from September to May in the last quarter near new moon, with a peak season during January and February. This is similar to spawning patterns for this species reported from Komodo National Park [13], Papua New Guinea [34] and Pohnpei, Micronesia [10]. The camouflage grouper spawns for three consecutive months (February to April) in Pohnpei, Micronesia [24]. The gonad development data show that Camouflage grouper seem to have a spawning peak in late January and April during the last quarter moon phase, which has been known as a partial spawner [21,24]. Based on the interviews with fishers (*n* = 174), camouflage groupers are occasionally seen in shallow water first in the last quarter period, and followed by tiger grouper and squaretail coral trout around the new moon.

### 4.3. The Minimum Size of Sexual Maturity

The live reef food fish trade (LRFFT) management in Indonesia needs to achieve sustainable fisheries, preferably using simple management approaches that could be efficiently applied by stakeholders [25]. Live fish destined for the LRFFT are generally weighed but only rarely is the length measured. Therefore, the mean length at first maturity (Lm_50_) of the three economically important groupers in this study (leopard coral trout, squaretail coral trout, and camouflage grouper) needed to be converted to weight (using the L-W relationship based on samples from this study). The size of the three studied grouper species was consistent with previous studies on the mean size at first sexual maturity [10,24,33].

In this study in Eastern Indonesia, leopard coral grouper achieve 50% size of sexual maturity (Lm_50_) at 37.7 TL cm, which is comparable to the finding in the Great Barrier Reef, Australia [33], where the fish reached first sexual maturity at 35 cm TL. The first size at sexual maturity of squaretail coral trout was the same with the squaretail coral trout found in Pohnpei, Micronesia [10] around 36.6 cm TL. Our findings of the first length maturity are for camouflage grouper are bigger for camouflage grouper compare with the conspecifics in Pohnpei, Micronesia [24], who reported that the first length of maturity for the species was 27 cm TL smaller than found in Eastern Indonesia up to 36.95 cm TL cm (Table 5). Based on the length–weight relationship at first length maturity of groupers, the average weight of market size groupers tends to be above the weight at first maturity. Nonetheless, the perception of size limits among live reef fish stakeholders needs to be adjusted to accommodate the data on reproductive biology, with a minimum weight limit of 800 g for the two coral trout species and at least 900 g for the camouflage grouper. An across the board size limit of 500 g is not appropriate for these three grouper species, as at this size very few individuals will have had an opportunity to reproduce. One challenge is that plate-sized (under 1000 g and preferably closer to 500 g) fish are most highly-priced in the LRFFT, especially for groupers [35].

The fishery and trade stakeholders would like a single size limitation on wild-caught live groupers in order to make the regulations relatively easy to comply with and also to enforce. However, in Indonesia there are many grouper species, with different life histories and more importantly size at sexual maturity. Some grouper species exploited in Indonesia mature well below the existing 500 g, while others would not mature at 1000 g or more [9]. Thus, any legislation will be a compromise, endeavoring to balance the biological requirements of the species exploited with the “art of the possible” in terms of real-world fisheries management in a still developing country.

Lessons learned from the size regulations for the Napoleon wrasse *Cheilinus undulatus* are instructive, and strike a cautionary note. Under Ministerial Regulation Number 37/KEPMEN-KP/2013, the export of Napoleon wrasse is limited to fish in the 1000 g to 3000 g range. To date, this regulation remains poorly, or indeed not, implemented [36]. There is a mismatch between the size allowed under the regulation and the available fishery resources (*C. undulatus* population structure and dynamics). Furthermore, size affects the profitability of the trade. These days, consumers in Hong Kong and other LRFFT destination countries prefer plate-sized fish (one per person) rather than a large fish to share. Consequently, for larger fish species, regulations based on fish reproduction will encounter market preference resistance and thus be difficult to implement. For the three groupers in this study, the average size of fish in the LRFFT in Eastern Indonesia is larger than the size at first sexual maturity, which is a positive finding. However, combining the data on LRFFT fisheries history with the Lm_50_ data in Table 1 indicates that the size at first maturity is lower (now) in areas where the LRFFT has been operating for longer. While it is not impossible that this could be a natural phenomenon or a chance result (especially as the sampling size was relatively low), this empirical correlation could be due to fishing pressure, and should be considered as a warning signal.

## 5. Conclusions

The exploitation of LRFFT species in Indonesia needs to be properly recorded, including volume and composition (species and size classes). Such data are vital for sustainable fisheries management, including assessing resources and management effectiveness. In a multi-species multi-scale fishery, this is no easy task. In the meanwhile, as a measure suitable or data-limited fisheries such as the LRFFT in groupers, size or weight limits are an attractive option [37]. While the current (unofficial and partial) weight limit of 500 g could have a positive impact, especially for smaller sized grouper species, the minimum weight for the three groupers species studied should be readjusted to 800 g per fish for both dead (fresh/frozen) and live fishes. Such a regulation is urgently needed for grouper fishery and trade sustainability, and would benefit both the wild fish stocks and the people who depend on them for their livelihoods [4]. In view of the high value and volume of the trade in *P. leopardus*, as well as the ease of recognizing this fish, it would be possible to begin with a specific regulation for this species. As data on grouper size at first maturity and spawning periods improve in quantity and quality, spatial and temporal closures (at spawning aggregation sites during peak spawning seasons) are recommended. Further research on sex change is urgently needed, especially if there are concerns that the rate of male removal may be too high and could potentially result in sperm limitation during spawning due to low male abundance [38]. The change in utilization, from a no-take zone to tourism zone (for dive activity), could potentially be an option for some permanent or temporary closures. Where spawning locations are relatively safe, they could be attractive for visitors, and such a use could contribute to the livelihoods of the surrounding community

## Figures and Tables

**Table 1:** Descriptive classification of maturity stages in female and male gonads of three grouper species based on histological characteristics, adapted from the classification for Nassau grouper (*Epinephelus striatus*) [27].

Gonad Development	Histological Description
Immature female	The whole gonad was small and compact; the gonads dominated with primary growth stage 1/2 oocytes (Figure 2A).
Mature resting female	The oocytes stage 1/2 and three presented with occasional early stage 4 oocytes (Figure 2B). This stage was mostly found between spawning seasons, but it was not possible to determine whether this stage of ovary had previously ovulated or was maturing for the first time. In this research, the mature resting female was dominated by large species.
Mature ripe female	All gonads dominated with oocytes stage 1/2, 3, 4, and vitellogenic oocytes present in various proportions, the ovarian wall may become thin (Figure 2C). There are only a few (if any) post-ovulatory follicles visible. At ovulation stage, hydrated eggs released from their follicular developed into the ovary lumen forming post-ovulatory follicles (that were only found around 24 h after ovulation) [32].
Post spawning female	Gonad contains degeneration of vitellogenic oocytes and 1/2 stages oocytes and lamella wall becomes thick (Figure 2D).
Bisexual	Gonad contained stages 1/2 oocytes and1/2 spermatocytes (immature bisexual) (Figure 3E); 3/4 oocytes and 3/4 spermatocytes (mature bisexual) appear together in similar amount (Figure 4F).
Immature male	All gonads were small and compact, contained a central lumen, and were dominated by seminiferous tubules. Scattered cysts of spermatocyte stage 1/2 were evident, but not common (Figure 3A). An occasional stage 1/2 oocyte was present.
Mature resting male	Gonad was dominated by early stages of spermatogenesis (i.e., spermatocyte stages 1 and 2) with scattered cysts of sperm increasingly evident as the spawning season approached. Occasional dispersed phase 1/2 oocytes were present. The testis lumen became smaller than in immature phase (Figure 3B).
Mature ripe male	Late stages of spermatogenesis were dominant and spread to fill almost all parts of the testis. Sperm occurred in expanded lobules, and spermatocyte stages 1 and 2 were relatively less abundant (Figure 3C).
Post spawning male	Most of the late stages of spermatogenesis have released. Spermatocyte stages 1 and 2 were relatively less abundant than the mature male phase. The gonad often had a thickened wall; there was space between the muscular wall and sperm (Figure 3D).

**Table 2:** Total sample of three groupers species per month and species during this study (*n* = 133). F refers to female, M refers to male, and T refers to transitional.

Species	Sexual Differentiation	2017					2018				Total
		May	Jun	Oct	Nov	Dec	Jan	Feb	Mar	Apr	
*Plectropomus leopardus*	F	0	4	4	21	25	7	9	0	0	70
	M	1	0	3	2	2	1	1	0	0	10
	T	1	0	0	2	1	1	0	0	0	5
*Plectropomus areolatus*	F	0	0	0	7	11	5	0	0	1	24
	M	0	0	0	1	1	0	0	0	0	2
	T	0	0	0	0	0	0	0	0	0	0
*Epinephelus polyphekadion*	F	0	0	3	6	1	7	0	0	1	18
	M	0	0	1	1	0	0	0	0	2	4
	T	0	0	0	0	0	0	0	0	0	0
Total											133

**Table 3:** Mean length at first sexual maturity of three grouper species from different areas in Eastern Indonesia, based on gonad samples collected during this study.

Species Name	Sampling Area	Lm_50_ (TL, cm)	Estimated Weight (g)	No. of Samples
*Plectropomus leopardus*	Kapoposang MTP	31.56	404.4	34
	Takabonerate NP	37.18	670.3	14
	Karas Islands	40.35	723.9	31
	Kei Island	47.78	1150.3	5
*Plectropomus areolatus*	Takabonerate NP	37.80	826.9	9
	Wakatobi NP	40.20	948.4	15
*Epinephelus polyphekadion*	Takabonerate NP	37.48	885.9	15
	Wakatobi NP	40.90	1058.4	5

MTP, Marine; Tourism Park. NP; National Park; **TL,** Total Length.

**Table 4:** Spawning aggregation periods according to fisher interviews: leopard coral trout, squaretail coral trout, and camouflage grouper in Eastern Indonesia. The aggregation sites are not listed here to protect the site.

Spawning Month/Location	Spawning Month			No. of Respondents
	*P. leopardus*	*P. areolatus*	*E. polyphekadion*	
Kapoposang MTP	Nov–Jan	-	-	30
Takabonerate NP	-	Sept–Feb	Jan–Apr	100
Wakatobi NP	-	Nov–Dec, Feb–May	Oct–Jan	48
Kei Islands	Oct–Dec	-	-	24
Karas Islands	-	Oct–Feb	Nov–Dec	26

**Table 5:** Representative of length at first maturity (Lm_50_) of three groupers species.

Species Name	Lm_50_ (cm)		Australia	References
	Indonesia	Pohnpei		
*Plectropomus leopardus*	37.7	-	35	[33]
*Plectropomus areolatus*	36.6	36.6	-	[10]
*Epinephelus polyphekadion*	36.9	27	-	[24]

## Appendix A

**Questionnaire for fishers**

Name:

Address (resident/not resident):

Age:

Last education:

1. When the first time you catch live reef fish?
2. Where you go to fishing live groupers (please mark in this map and tell me what kind of fish you will get there)?
3. Is the place the same as your first time fishing live groupers? If not, please mark where the first time are you fishing live groupers?
4. Did you ever see the fish aggregation? What kind of fish? How depth and when they aggregate? If any, please mark the place?
5. When do you see, the fish came to aggregate? Please tell us the month and moon phase?
6. Is there any missing place (a long time ago were many groupers there, and now the groupers were gone), if any, please mark the place?

Additional information:
